# A New and Valuable Surgical Instrument, of Texas Invention

**Published:** 1889-09

**Authors:** 


### A NEW AND VALUABLE SURGICAL INSTRUMENT OF TEXAS INVENTION.

Dr. R. T. Knox, of Gonzales, has invented an instrument for dilating the os-uteri, which seems to possess many points of advantage over similar instruments on the market. Tieman & Co. are manufacturing it, and, we understand, will soon offer it to the profession. They speak very highly of the invention.

"Necesssity" may be said, truly, to have been the "mother of" this invention. Dr. Knox practices at a point remote from "medical centers," and where he cannot, like "city physicians," have access to an *armamentarium chirurgicum*, or call in some specialist when an unusual difficulty is presented. On one occasion, he was called to a case of convulsions, "occurring at regular periods, with increased suffering. It was a case of retained menses, and for which much medicine had been given, with only partial and temporary relief, until it was feared occlusion had taken place. The agony was very great, and relief in some shape was imperative. Beyond the reach of assistance or counsel, I

proposed mechanical relief," says the doctor, in a letter accompanying the model sent us. "It was accepted, and dilation was made by improvising a dilator, using a pair of long bullet forceps, (reversing the motion). The stricture was broken, or rigidity (as the case may be) was overcome; smiles took the place of tears, and all was lovely, as the flow came on copiously, —very dark." This suggested to Dr. Knox the instrument under consideration.

It is a dilator and drainage tube combined. It consists of two blades with double curve, so that when the handles are approximated the points diverge; when the thumb and finger rings are in apposition, the points are at their widest angle, on the principle of a glove-stretcher. The point consists of two hollow half-cylinders, about four inches in length, and which, when put with their flat surfaces together, form a hollow cylinder tapering to a point from the butt, where they screw on to the blades, or handles, this butt being about 3-5 of an inch in diameter. This tube admits of the escape of pent up fluid, as in the case cited, acting as a drainage tube, or of the introduction of washes or other medication by means of syringe. The tube is graduated, and the operator knows, by a corresponding scale on the blades, just the extent of the dilation at any stage of the operation, and the extent to which the instrument has penetrated. The instrument is made with two sets of tubes, or parts, one straight, the other slightltly curved, and as they are detachable, either can be used at pleasure. This also renders them easily cleansed.

The instrument undoubtedly has merit, and will be a valuable addition to the surgeon's and gynecologist's outfit.

